# Medicare Part B Spending on Macular Degeneration Treatments Associated With Manufacturer Payments to Ophthalmologists

**DOI:** 10.1001/jamahealthforum.2023.2951

**Published:** 2023-09-08

**Authors:** Sean R. Dickson, Katelyn E. James

**Affiliations:** 1 West Health Policy Center, Washington, DC; 2 now with AHIP, Washington, DC

## Abstract

**Importance:**

Age-related macular degeneration (ARMD) therapies aflibercept and ranibizumab are among the highest-cost Medicare Part B drugs, even though off-label use of lower-cost bevacizumab is clinically noninferior. Payments from manufacturers of these ARMD therapies to ophthalmologists are hypothesized to be factors in ophthalmologists' therapeutic choice, controlling for ophthalmologist and patient characteristics.

**Objective:**

To assess the association between manufacturer payments to ophthalmologists and choice of ARMD treatment as well as to identify ophthalmologist-level characteristics associated with prescribing lower-cost ARMD therapies.

**Design, Setting, and Participants:**

This retrospective cross-sectional study of longitudinal (2013-2019) Medicare Part B data was conducted from December 2021 to December 2022. Ophthalmologists prescribing aflibercept (manufactured by Regeneron Pharmaceuticals Inc), rabinizumab, or bevacizumab (both manufactured by Genentech Inc) for ARMD treatment of Medicare Part B beneficiaries were included. Data on manufacturer payments to ophthalmologists were obtained from the Open Payments database.

**Main Outcomes and Measures:**

The primary outcome was the percentage of bevacizumab prescribed by ophthalmologists among all ARMD therapies. Regression analysis assessed variation in bevacizumab prescribing by acceptance of manufacturer payments as well as by ophthalmologist and patient characteristics. Ophthalmologist characteristics were duration of practice and Medicare Administrative Contractor region, and patient characteristics were aggregated at the ophthalmologist level and included mean beneficiary age, percentage of dual-eligible beneficiaries, mean beneficiary risk score, and percentage of White beneficiaries. Savings were estimated by projecting the change in bevacizumab use had ophthalmologists not accepted manufacturer payments, controlling for all ophthalmologist and patient characteristics and comparing with observed use and costs.

**Results:**

A total of 21 584 ophthalmologists (18 489 males [85.7%]) were included. Ophthalmologists who accepted manufacturer payments were significantly less likely to prescribe bevacizumab (28.0% [95% CI, 24.6%-42.5%] of patients) compared with those who did not accept manufacturer payments (45.8% [95% CI, 44.5%-47.1%]). Ophthalmologists who saw dual-eligible beneficiaries had greater bevacizumab prescribing (50.0% [95% CI, 40.6%-68.3%] in the highest quartile vs 36.1% [95% CI, 33.5%-38.8%] in the lowest quartile; β coefficient, 0.139; *P* < .001), while those who saw patients with higher mean beneficiary risk scores had lower bevacizumab use (38.0% [95% CI, 23.7%-44.1%] in the highest quartile vs 48.2% [95% CI, 45.5%-50.8%] in the lowest quartile; β coefficient, −0.102, *P* < .001). Had ophthalmologists who accepted manufacturer payments prescribed ARMD drugs as those who did not accept payments, Medicare spending on these treatments would have been $642 779 703.08 lower from 2013 to 2019, a 2.0% savings.

**Conclusions and Relevance:**

Results of this cross-sectional study suggest that drug manufacturer payments to ophthalmologists were associated with selection of higher-cost therapies for ARMD, which is a factor in increased Medicare and patient spending. Development of manufacturer payment models that encourage ophthalmologists to choose lower-cost therapies are needed.

## Key Points

**Question:**

Do ophthalmologists who accept payments from manufacturers of age-related macular degeneration (ARMD) drugs prescribe higher-cost therapies?

**Findings:**

In this cross-sectional study of 2013 to 2019 Medicare Part B data including 21 584 ophthalmologists, ophthalmologists who accepted manufacturer payments from ARMD drug manufacturers were significantly less likely to prescribe a lower-cost therapy (28.0%) than ophthalmologists who did not accept payments (45.8%), controlling for ophthalmologist and patient characteristics. Had ophthalmologists who accepted payments prescribed as those who did not accept payments, Medicare spending on ARMD treatments over the study period would have been more than $642 million lower.

**Meaning:**

Findings of this study suggest that manufacturer payments to ophthalmologists were associated with higher-cost therapeutic choice for ARMD as well as increased Medicare and patient spending.

## Teaser

This cross-sectional study evaluates whether research and nonresearch payments from drug manufacturers play a role in opthalmologists’ selection of therapies for age-related macular degeneration.

## Introduction

Three anti–vascular epithelial growth factor (VEGF) treatments are equally recommended by the American Academy of Ophthalmology for age-related macular degeneration (ARMD).^1,2,3^ A progressive blinding disease that affects approximately 19.5 million people in the US, ARMD is the leading cause of blindness in adults 50 years or older.^4^ Three anti–VEGF medications have been used for first-line of treatment of ARMD, as preferred by the American Academy of Ophthalmology: bevacizumab, ranibizumab, and aflibercept.^1^ These anti–VEGF treatments were substantially different in price in 2020: on average, off-label use of bevacizumab (Avastin; Genentech Inc) cost $70.86 per Medicare claim, while ranibizumab (Lucentis; Genentech Inc) cost $333.55 and aflibercept (Eylea; Regeneron Pharmaceuticals Inc) cost $921.65.^5^ These cost differences have implications for both the Medicare program and beneficiaries, as these patients are liable for 20% of the treatment cost.^6,7^ Aflibercept was the second most expensive drug by total spending for the Medicare Part B program in 2020, with more than $3 billion in annual costs, while ranibizumab was the sixth most expensive drug by total spending, costing more than $1 billion; spending on aflibercept and ranibizumab together was more than 10% of the total Medicare Part B drug spending for 2020.^8^ Prior work has modeled the savings that could be achieved from encouraging ophthalmologists to choose the lowest-cost treatment option for ARMD.^9^ In this study, we explored the ophthalmologist-level characteristics associated with choice of higher-cost vs lower-cost ARMD treatment. We then estimated the increase in Medicare spending associated with the selection of higher-cost treatment by ophthalmologists who received payments from the manufacturers of aflibercept and ranibizumab (Regeneron Pharmaceuticals Inc and Genentech Inc, respectively), controlling for other ophthalmologist characteristics.

In 2004, bevacizumab was approved by the US Food and Drug Administration (FDA) to treat various forms of cancer, particularly targeting tumor angiogenesis for metastatic colorectal cancer, but it was quickly adapted by ophthalmologists as an off-label treatment for ARMD.^10,11^ In 2006, ranibizumab was approved by the FDA for treatment of neovascular ARMD.^12^ Both bevacizumab and ranibizumab are manufactured by Genentech Inc, and both products are derived from the same murine antibody to VEGF.^13,14^ Ranibizumab is a fragment of bevacizumab and has higher binding affinity, but in clinical applications, both products have equal success in treating ARMD.^12^ When used for treatment of ARMD, bevacizumab is repackaged from its oncology vial size by specialist pharmacies for intravitreal injection.^13^ In 2011, aflibercept received FDA approval for treatment of ARMD.^15^

Under the Medicare Part B program, all health care practitioners administering drugs are reimbursed based on the average sales price (ASP) of the drug, plus a 6% add-on payment (which was limited to 4.3% from 2013-2024 due to a federal budget sequester).^16,17^ To ensure that a practitioner participating in Medicare is adequately reimbursed for a particular drug, Medicare establishes a separate payment code for each drug with its own ASP. Because the ASP is a mean price, in theory, some practitioners may purchase the drug above the ASP due to volume and other discounts negotiated by other practitioners; therefore, the add-on payment helps ensure that practitioners are not underreimbursed for their drug acquisition costs.^17,18,19^ In practice, however, this percentage-based add-on payment creates an incentive to prefer a higher-cost product over an equivalent lower-cost product given that the practitioner gains greater profit, an incentive that has been documented in numerous government reports.^9,20,21^ For the 3 anti–VEGF drugs, the add-on payment generates more than twice the ophthalmologist profit for choosing aflibercept or ranibizumab over bevacizumab (Table 1).^5^

**Table 1.** Medicare Part B Costs and Add-On Payments for Age-Related Macular Degeneration Therapies^a^

Therapy	Mean spending per unit, $^b^	Mean units per claim^c^	Mean spending per claim, $	Mean No. of claims per beneficiary per year^c^	Mean annual spending per beneficiary, $	Mean annual add-on payment per beneficiary, $
Bevacizumab (Avastin)	76.32	14	1068.48	3.9	4167.07	171.80
Aflibercept (Eylea)	923.56	2	1847.12	5.0	9235.60	380.76
Ranibizumab (Lucentis)	333.55	5	1667.75	5.1	8505.52	350.66

^a^ Under the Medicare Part B program, ophthalmologists are reimbursed at the average sales price (ASP) for a drug plus an add-on payment (6% of the ASP by statute but was limited to 4.3% from 2013-2024 due to a federal budget sequester). The mean annual add-on payment is calculated as the difference between mean annual spending and mean annual spending divided by 1.043, as the mean annual spending already includes the add-on amount.

^b^ Mean spending per unit values were from the 2020 Medicare Part B dashboard.

^c^ Mean units per claim and mean claims per beneficiary per year were calculated from Medicare Physician & Other Practitioners utilization data from 2020 for ophthalmologists.

Previous research has documented the overall variation in use of bevacizumab, ranibizumab, and aflibercept, demonstrating historically higher use of bevacizumab over ranibizumab, even though ranibizumab had greater ARMD treatment spending.^22,23^ Black Medicare beneficiaries were more likely to receive bevacizumab over ranibizumab, while higher-income beneficiaries were more likely to receive ranibizumab.^24,25^ After the introduction of aflibercept, ophthalmologists, who predominantly administered ranibizumab over bevacizumab, were more likely to shift to aflibercept.^15^ Ophthalmologists who received consulting or research payments from Regeneron Pharmaceuticals Inc and Genentech Inc were more likely to administer aflibercept and ranibizumab over bevacizumab compared with ophthalmologists who did not receive any manufacturer payments.^26,27^

Rather than broadly describe overall patterns in ARMD-drug prescribing, this study aimed to assess the association between manufacturer payments to ophthalmologists and choice of ARMD treatment as well as to identify ophthalmologist-level characteristics associated with prescribing lower-cost ARMD therapies. This approach has 2 key policy implications. First, it establishes a method for evaluating individual ophthalmologist practice variations from the norm, which can be used to identify outlier ophthalmologists who may be overprescribing higher-cost therapies. Second, it creates a framework for developing value-based payment models for ARMD treatment, identifying high-quality, lower-cost ophthalmologists who can serve as models for innovative reimbursement methodologies. Because the 3 ARMD drugs that we assessed in the study are widely regarded as interchangeable, this approach can highlight variations in practice pattern that are associated with economic or other factors rather than clinical necessity. As an initial step, we identified a model group of ophthalmologists (those who did not accept manufacturer payments) and projected the savings had the ophthalmologists who accepted manufacturer payments practiced in the same way as the model group, controlling for all other ophthalmologist characteristics. More rigorous payment reforms, however, could identify a more limited model group, with greater use of low-cost therapies, and could establish payment rates that reflect the therapeutic distribution in this model population.

## Methods

Per the decision guidance of the US Department of Health and Human Services, this cross-sectional study was exempt from institutional review board approval and informed consent because it used only publicly available data. We followed the Strengthening the Reporting of Observational Studies in Epidemiology (STROBE) reporting guideline.

### Data Sources

We obtained data from the 2013 to 2019 Medicare Physician & Other Practitioners files available from the Centers for Medicare & Medicaid Services (CMS).^28,29^ These data are organized by National Provider Identifiers (NPIs) and contain summaries of the total number of claims, units, and beneficiaries receiving a particular service from a particular ophthalmologist. Using these NPIs, we linked ophthalmologist-level utilization data to the Medicare Doctors and Clinicians National Downloadable File to estimate the number of years an ophthalmologist has been practicing based on the ophthalmologist’s year of graduation from medical school.^30^ We linked these data to the CMS Open Payments data to identify ophthalmologists who received payments from Regeneron Pharmaceuticals Inc and Genentech Inc, matching ophthalmologists by name and location, consistent with methods in other publications.^9,25^ Open Payments contains data on any manufacturer payment or transfer of value to physicians and other practitioners participating in Medicare. These manufacturer payments include compensation, research funding, travel, meals, entertainment, and long-term device loans among other categories.^31^

### Study Sample

Ophthalmologists were included in the study if they were identified as ophthalmologists and had billed Medicare for any of the 3 drugs of interest. We identified claims for each of the drugs using the Healthcare Common Procedure Coding System (HCPCS) codes. Physician bills were paid under the code J2778 for ranibizumab, code J0178 for aflibercept, and code J0935 for bevacizumab.^32,33,34^ During the study period, some off-label bevacizumab use was billed under different HCPCS codes across different Medicare Administrative Contractor (MAC) regions based on local guidance. We performed a systematic review of all MAC billing guidance to identify local codes for bevacizumab billing and used codes J3490 (for unclassified drugs), J3590 (unclassified biologics), J9777 (for compounded drugs), and J9035 (bevacizumab injection code) for each MAC and period when those codes were indicated for use.^35^ Because some of these codes were not specific to bevacizumab, we considered the use billed under these codes to be for bevacizumab only if the per-unit price was within the 10th to 90th percentile of per-unit prices billed under bevacizumab-specific codes for that year. In a sensitivity analysis, we excluded ophthalmologists with these nonbevacizumab-specific codes.

### Statistical Analysis

Data were organized as longitudinal panel data with the ophthalmologist NPI as the panel variable, and time was measured by year. The dependent variable was the percentage of total claims for bevacizumab (bevacizumab claims/[bevacizumab claims + aflibercept claims + ranibizumab claims] 100×). The independent variables were each ophthalmologist’s duration of practice or number of years since graduation from medical school (by quartiles of 0-12, 13-21, 22-30, and 31-68 years); MAC regions; an indicator for whether bevacizumab was billed under a nonbevacizumab-specific code; and an indicator for whether any payment from either manufacturer (Regeneron Pharmaceuticals Inc or Genentech Inc) was accepted, as well as the following patient characteristics aggregated at the ophthalmologist level: annual mean beneficiary age (by quartiles of 62-75, 76, 77, and 78-84 years), mean beneficiary risk score (by quartiles of 0.76-1.28, 1.29-1.44, 1.45-1.58, and 1.59-3.40 points), percentage of beneficiaries who were dual eligible for Medicare and Medicaid (by quartiles of 0%-8.65%, 8.66%-12.56%, 12.57%-19.34%, and 19.35%-97.2%), and the percentage of White beneficiaries (by quartiles of 0%-75%, 76%-86%, 87%-92%, and 93%-100%). White beneficiary patient share was included based on research finding that variations in prescribing were associated with the race and ethnicity of beneficiaries.^15,25,36^ Given the suppression requirements of Medicare data, we were able to accurately use only White and other-than-White patient characterizations in the model. Race and ethnicity summary data were as reported in Medicare utilization data and included American Indian or Alaska Native, Asian or Native Hawaiian and Pacific Islander, Black, Hispanic, White, and other.

In a separate regression analysis, 2 indicator variables were used to assess manufacturer payments: whether the ophthalmologist accepted research-only funding from Regeneron Pharmaceuticals Inc or Genentech Inc or whether the ophthalmologist accepted any nonresearch payments (including consulting fees, speaking fees, and honoraria; these ophthalmologists may also have accepted research funding) from either company. Mean beneficiary characteristics were for all beneficiaries seen by the ophthalmologist, not just the beneficiaries treated for ARMD, and reflected the overall practice environment for each ophthalmologist.

The primary outcome was the percentage of bevacizumab prescribed by ophthalmologist among all macular degeneration therapies. We used a between-effects model rather than a fixed-effects model for several reasons. First, the between-effects model considered the differences in prescribing patterns across ophthalmologists rather than changes in prescribing patterns associated with changes in the independent variables for an individual ophthalmologist. For example, the between-effects model allowed us to compare the relative difference in prescribing patterns among ophthalmologists who saw a higher percentage of beneficiaries with race and ethnicity other than White vs ophthalmologists who saw a lower percentage. A fixed-effects model, in contrast, would consider the year-over-year change in prescribing patterns for each ophthalmologist based on the change in percentage of beneficiaries with race and ethnicity other than White who were seen by that ophthalmologist. Because we did not expect patient demographic characteristics to substantially vary from year to year for a given ophthalmologist, we used the between-effects model. Second, we hypothesized that prior acceptance of manufacturer payments may affect prescribing in subsequent years even if a payment is not accepted in that year. Because we could not determine whether manufacturer payments were made prior to the study period, we used a periodwide indicator variable for ever accepting payments rather than assuming that no ophthalmologist had accepted payments prior to the study period. Because the between-effects model used the mean value for each variable for each ophthalmologist during the study period, we avoided autocorrelation concerns and accounted for any dropout, as only periods with data were included in the mean. Furthermore, we assumed that any temporal outcomes were consistent across ophthalmologists during the study period. Regression analysis results were presented as estimated mean marginal effect size at quartile values for each beneficiary and ophthalmologist characteristic for the dichotomous independent variables, estimating the percentage of bevacizumab that an ophthalmologist with those characteristics would prescribe. We also reported β coefficients, which represent the percentage-point differences in bevacizumab use from the index group (which generally included the first quartile in each variable, but the index group differed according to the covariate). Two-tailed *P* < .05 was considered to be statistically significant.

We estimated the increased Medicare spending associated with manufacturer payments to ophthalmologists by projecting the therapeutic utilization mix for ophthalmologists who accepted manufacturer payments as though they had not accepted payments, controlling for all characteristics of the practitioner-beneficiary mix. We then subtracted the hypothetical spending associated with this projected therapeutic mix from the observed spending for each ophthalmologist.

Data consolidation was performed in R, version 4.1 (R Foundation for Statistical Computing). Data analysis was completed with Stata, version 16 (StataCorp LLC), from December 2021 to December 2022.

## Results

The study included 21 584 ophthalmologists from 2013 to 2019, of whom 3095 were females (14.3%) and 18 489 were males (85.7%). Characteristics of ophthalmologists who accepted (5499 [25.5%]) or did not accept (16 085 [74.5%]) manufacturer payments are presented in Table 2. Bevacizumab use across all ophthalmologists decreased from 39.9% of claims in 2013 to 31.2% in 2019. The percentage of ophthalmologists accepting payments from Regeneron Pharmaceuticals Inc or Genentech Inc varied over the period, from a low of 15.0% in 2017 to a high of 34.3% in 2015, averaging 25.7% over the study period.

**Table 2.** Ophthalmologist Characteristics by Acceptance of Manufacturer Payments From 2013 to 2019^a^

Characteristic	Ophthalmologists, No. (%)			*P* value
	Overall	Did not accept payments	Accepted any payments^b^	
Unique ophthalmologists	21 584 (100)	16 085 (74.5)	5499 (25.5)	NA
Accepted only research payments	NA	NA	641 (3.0)	NA
Mean (SD) duration of practice, y	21.7 (11.0)	21.6 (11.3)	22.0 (10.2)	.05
Mean (SD) beneficiary age, y	75.9 (2.1)	75.7 (2.2)	76.3 (1.9)	<.001
Dual-eligible beneficiaries	3 125 303 (15.4)	2 354 011 (19.5)	771 292 (13.7)	<.001
Beneficiaries with race and ethnicity other than White^c^	817 083 (4.7)	583 470 (4.7)	233 613 (4.7)	.48
Mean (SD) beneficiary risk score	1.45 (0.3)	1.44 (0.3)	1.47 (0.2)	<.001
Beneficiaries in 2013	445 106 (100)	286 864 (64.4)	158 242 (35.6)	
Bevacizumab use	177 597 (39.9)	129 375 (45.1)	48 264 (30.5)	<.001
Ranibizumab use	153 561 (34.5)	86 059 (30.0)	67 411 (42.6)	<.001
Aflibercept use	113 502 (25.5)	71 142 (24.8)	42 408 (26.8)	<.001
Beneficiaries in 2019	636 351 (100)	447 833 (70.4)	188 518 (29.6)	
Bevacizumab use	198 542 (31.2)	156 293 (34.9)	42 228 (22.4)	<.001
Ranibizumab use	45 069 (22.7)	81 058 (18.1)	63 530 (33.7)	<.001
Aflibercept use	91 528 (46.1)	210 481 (47.0)	82 948 (44.0)	<.001

Abbreviation: NA, not applicable.

^a^ Ophthalmologist characteristics and utilization data were from the Medicare Physician & Other Practitioners files. Payment data were from the Centers for Medicare & Medicaid Services Open Payments database.

^b^ Any payments included both research funding and nonresearch payments, including consulting fees, speaking fees, and honoraria.

^c^ Race and ethnicity were reported in Medicare utilization data; other than White included American Indian or Alaska Native, Asian or Native Hawaiian and Pacific Islander, Black, Hispanic, and other.

All independent variables except for beneficiary race and ethnicity were significant at the *P* < .05 level in the model (Table 3). Increasing mean beneficiary age was associated with lower bevacizumab use (35.2% [95% CI, 20.4%-41.6%] in the highest quartile vs 45.8% [95% CI, 43.7%-47.9%] in the lowest quartile, a 10.6 percentage-point difference; β coefficient, −0.106, *P* < .001), as was increasing mean beneficiary risk score (38.0% [95% CI, 23.7%-44.1%] in the highest quartile vs 48.2% [95% CI, 45.5%-50.8%] in the lowest quartile; β coefficient, −0.102, *P* < .001). However, increasing mean percentage of dual-eligible beneficiaries was associated with greater bevacizumab use (50.0% [95% CI, 40.6%-68.3%] in the highest quartile vs 36.1% [95% CI, 33.5%-38.8%] in the lowest quartile; β coefficient, 0.139; *P* < .001) as was a longer duration of practice (42.2% [95% CI, 39.3%-51.2%] in the highest quartile vs 40.8% [95% CI, 38.6%-43.1%] in the lowest quartile; β coefficient, 0.013; *P* < .001). Statistically significant differences were also observed by MAC region, with the highest utilization in region JH (61.1%; 95% CI, 18.9%-117.1%) and the lowest utilization in region J5 (10.4%; 95% CI, 3.2%-16.6%).

**Table 3.** Estimated Bevacizumab Use by Ophthalmologist Characteristics as a Percentage of Age-Related Macular Degeneration Prescriptions Following Between-Effects Panel Regression From 2013 to 2019

Characteristic	Estimated values by quartile	Estimated bevacizumab use (95% CI)	Regression coefficient, β^a^	*P* value
Accepted any payments from manufacturers	Yes	28.0 (24.6-42.5)	–0.178	<.001
	No	45.8 (44.5-47.1)	NA	
Percentage of White beneficiaries, %^b^	0-75	40.8 (38.2-43.5)	NA	
	76-86	38.7 (33.0-40.1)	–0.022	.23
	87-92	42.9 (41.1-48.9)	0.021	.29
	93-100	42.2 (39.5-47.6)	0.013	.52
Mean beneficiary risk score, points^b^	0.76-1.28	48.2 (45.5-50.8)	NA	
	1.29-1.44	41.4 (30.8-44.3)	–0.068	.001
	1.45-1.58	37.6 (22.9-44.0)	–0.106	<.001
	1.59-3.40	38.0 (23.7-44.1)	–0.102	<.001
Mean beneficiary age, y^b^	62-75	45.8 (43.7-47.9)	NA	
	76	41.0 (32.7-42.2)	–0.048	.010
	77	38.4 (27.3-42.1)	–0.074	<.001
	78-84	35.2 (20.4-41.6)	–0.106	<.001
Duration of practice, y	0-12	40.8 (35.4-39.4)	NA	
	13-21	38.7 (38.4-45.3)	0.035	.032
	22-30	42.8 (40.0-51.7)	0.058	<.001
	31-68	42.2 (39.3-51.2)	0.060	<.001
Percentage of dual-eligible beneficiaries, %^b^	0-8.65	36.1 (33.5-38.8)	NA	
	8.66-12.56	39.5 (39.2-46.6)	0.034	.08
	12.57-19.34	39.6 (38.9-46.0)	0.028	.12
	19.35-97.20	50.0 (40.6-68.3)	0.139	<.001
Annual Medicare beneficiaries, No.	11-45	49.1 (46.7-51.5)	NA	
	46-123	39.9 (27.3-45.6)	–0.092	<.001
	124-263	39.8 (26.8-45.4)	–0.093	<.001
	264-1514	36.7 (20.1-45.0)	–0.124	<.001
Use of nonbevacizumab-specific coding	Yes	52.3 (52.2-71.2)	0.119	.001
	No	40.4 (39.3-41.5)	NA	
MAC region^c^	J15	10.9 (5.8-16.1)	NA	
	J5	10.4 (3.2-16.6)	–0.005	.88
	J6	51.0 (17.1-54.1)	0.401	<.001
	J8	7.44 (2.7-10.5)	–0.035	.30
	JE	43.6 (17.1-83.1)	0.326	<.001
	JF	44.4 (17.1-83.9)	0.334	<.001
	JH	61.1 (18.9-117.1)	0.501	<.001
	JJ	52.0 (17.7-99.9)	0.411	<.001
	JK	42.8 (16.8-80.5)	0.319	<.001
	JL	50.5 (16.9-96.1)	0.396	<.001
	JM	42.0 (17.4-79.5)	0.310	<.001
	JN	14.6 (11.7-24.5)	0.037	.25

Abbreviations: MAC, Medicare Administrative Contractor; NA, not applicable.

^a^ Regression coefficients can be interpreted as the percentage-point change in bevacizumab use among all age-related macular degeneration therapies vs the index group (which generally included the first quartile in each variable, but the index group differed according to the covariate).

^b^ Percentage of White beneficiaries, mean beneficiary age, percentage of dual-eligible beneficiaries, and mean beneficiary risk score were ophthalmologist-level values for all Medicare beneficiaries seen by each ophthalmologist.

^c^ MAC regions were as follows: J5, Wisconsin Physician Services or WPS (Iowa, Kansas, Missouri, Nebraska); J6, National Government Services or NGS (Illinois, Minnesota, Wisconsin); J8, WPS (Indiana, Michigan); J15, CGS Administrators (Kentucky, Ohio); JE, Noridian (California, Hawaii, Nevada); JF, Noridian (Alaska, Arizona, Idaho, Montana, North Dakota, Oregon, South Dakota, Utah, Washington, Wyoming); JH, Novitas (Arkansas, Colorado, Louisiana, Mississippi, New Mexico, Oklahoma, Texas); JJ, Palmetto (Alabama, Georgia, Tennessee); JK, NGS (Connecticut, Maine, Massachusetts, New Hampshire, New York, Rhode Island, Vermont); JL, Novitas (Delaware, Maryland, New Jersey, Pennsylvania, Washington, DC); JM, Palmetto (North Carolina, South Carolina, Virginia, West Virginia); JN, First Coast Service Options (Florida).

Accepting payments from Regeneron Pharmaceuticals Inc or Genentech Inc was associated with lower bevacizumab use. Among ophthalmologists who accepted manufacturer payments, estimated bevacizumab use was 28.0% (95% CI, 24.6%-42.5%), while ophthalmologists who did not accept manufacturer payments were estimated to have prescribed bevacizumab 45.8% (95% CI, 44.5%-47.1%) of the time. In a separate regression model, accepting only research funding from Regeneron Pharmaceuticals Inc or Genentech Inc was associated with lower bevacizumab prescribing than accepting any nonresearch payments (these ophthalmologists may also have received research funding). Ophthalmologists who accepted only research funding were estimated to have prescribed bevacizumab 18.9% (95% CI, 11.6%-65.4%) of the time, a 26.9–percentage-point decrease compared with ophthalmologists who did not accept any manufacturer payments (45.8% [95% CI, 44.5%-47.1%]; β coefficient, –0.269; *P* < .01); ophthalmologists who accepted any nonresearch payments were estimated to have prescribed bevacizumab 28.3% (95% CI, 7.3%-42.5%) of the time (β coefficient, –0.176; *P* < .001); regression parameters for all other variables were consistent with the baseline model. Similar results for all variables were observed in the sensitivity analysis, which excluded ophthalmologists who billed for off-label bevacizumab using nonbevacizumab-specific billing codes (Table 4).

**Table 4.** Sensitivity Analysis Excluding Ophthalmologists Who Used Nonspecific Reimbursement Codes for Bevacizumab From 2013 to 2019

Characteristic	Estimated values by quartile	Estimated bevacizumab use (95% CI)	Regression coefficient, β^a^	*P* value
Accepted any payments from manufacturers	Yes	27.1 (23.7-40.8)	–0.171	<.001
	No	44.2 (42.9-45.5)	NA	
Percentage of White beneficiaries, %^b^	0-75	39.6 (36.9-42.3)	NA	
	76-86	37.6 (31.9-39.1)	–0.020	.26
	87-92	41.7 (39.8-47.7)	0.021	.30
	93-100	40.5 (37.2-45.5)	0.008	.69
Mean beneficiary risk score, points^b^	0.76-1.28	47.4 (44.8-50.1)	NA	
	1.29-1.44	39.6 (27.9-43.6)	–0.078	<.001
	1.45-1.58	36.6 (21.6-43.3)	–0.109	<.001
	1.59-3.40	36.2 (20.9-43.3)	–0.112	<.001
Mean beneficiary age, y^b^	62-75	44.0 (41.9-46.1)	NA	
	76	39.4 (31.2-40.3)	–0.046	.01
	77	37.4 (27.0-40.3)	–0.066	.001
	78-84	34.6 (21.0-39.8)	–0.094	<.001
Duration of practice, y	0-12	35.7 (33.7-37.7)	NA	
	13-21	39.7 (38.9-46.9)	0.040	.01
	22-30	41.7 (38.8-50.8)	0.060	<.001
	31-68	42.5 (38.5-52.3)	0.068	<.001
Percentage of dual-eligible beneficiaries, %^b^	0-8.65	34.2 (31.5-36.8)	NA	
	8.66-12.56	38.0 (37.9-45.6)	0.039	.04
	12.57-19.34	37.5 (37.2-44.4)	0.033	.07
	19.35-97.2	49.6 (38.7-69.5)	0.154	<.001
Annual Medicare beneficiaries, No.	11-45	47.6 (45.2-50.1)	NA	
	46-123	38.8 (26.6-44.4)	–0.088	<.001
	124-263	38.0 (24.7-43.9)	–0.096	<.001
	264-1514	35.3 (18.8-43.5)	–0.123	<.001
MAC region^c^	J15	6.3 (0.9-11.6)	NA	
	J5	9.9 (6.6-20.4)	0.036	.30
	J6	50.9 (12.6-100.9)	0.446	<.001
	J8	6.9 (0.8-14.3)	0.006	.85
	JE	40.1 (12.5-80.3)	0.339	<.001
	JF	40.6 (12.6-81.2)	0.343	<.001
	JH	60.8 (12.4-121.4)	0.545	<.001
	JJ	51.8 (15.2-104.3)	0.455	<.001
	JK	41.8 (12.2-83.3)	0.355	<.001
	JL	50.5 (12.4-100.9)	0.442	<.001
	JM	38.9 (12.9-78.2)	0.327	<.001
	JN	13.8 (12.7-27.8)	0.075	.02

Abbreviations: MAC, Medicare Administrative Contractor; NA, not applicable.

^a^ Regression coefficients can be interpreted as the percentage point change in bevacizumab use among all age-related macular degeneration therapies vs the index group (which generally included the first quartile in each variable, but the index group differed according to the covariate).

^b^ Percentage of White beneficiaries, mean beneficiary age, percentage of dual-eligible beneficiaries, and mean beneficiary risk score are ophthalmologist-level values for all Medicare beneficiaries seen by that ophthalmologist.

^c^ MAC regions were as follows: J5, Wisconsin Physician Services or WPS (Iowa, Kansas, Missouri, Nebraska); J6, National Government Services or NGS (Illinois, Minnesota, Wisconsin); J8, WPS (Indiana, Michigan); J15, CGS Administrators (Kentucky, Ohio); JE, Noridian (California, Hawaii, Nevada); JF, Noridian (Alaska, Arizona, Idaho, Montana, North Dakota, Oregon, South Dakota, Utah, Washington, Wyoming); JH, Novitas (Arkansas, Colorado, Louisiana, Mississippi, New Mexico, Oklahoma, Texas); JJ, Palmetto (Alabama, Georgia, Tennessee); JK, NGS (Connecticut, Maine, Massachusetts, New Hampshire, New York, Rhode Island, Vermont); JL, Novitas (Delaware, Maryland, New Jersey, Pennsylvania, Washington, DC); JM, Palmetto (North Carolina, South Carolina, Virginia, West Virginia); JN, First Coast Service Options (Florida).

Had ophthalmologists who accepted manufacturer payments seen the same therapeutic mix and prescribed the same way as similarly situated ophthalmologists who did not accept manufacturer payments, Medicare spending over the study period would have been $642 779 703.08 lower from 2013 to 2019, a 2.0% reduction in spending on ARMD treatments (Table 5). Bevacizumab use would have been 3.2% higher (40.3%) than observed (37.1%) over the study period, with mean per-beneficiary savings of $5231.35 per year.

**Table 5.** Estimated Increase in Medicare Part B Spending on Age-Related Macular Degeneration (ARMD) Treatments Attributable to Higher-Cost Prescribing Associated With Accepting Manufacturer Payments From 2013 to 2019

Year	Percentage of observed bevacizumab use	Percentage of estimated bevacizumab use	Per-beneficiary difference in spending between bevacizumab and nonbevacizumab use, $^a^	Estimated increase in Medicare spending, $	Percentage of Medicare Part B spending on ARMD treatments
2013	39.9	41.7	4177.47	32 592 590.87	0.9
2014	40.0	43.0	4694.64	89 399 963.61	2.4
2015	38.3	42.5	4575.73	101 210 464.21	2.4
2016	39.2	44.9	4994.77	159 053 475.21	3.4
2017	36.7	37.5	5708.26	28 415 739.91	0.6
2018	37.0	40.7	6009.00	141 782 386.63	2.7
2019	31.2	33.6	5926.84	90 325 082.64	1.6
Total	37.1	40.3	5231.35	642 779 703.08	2.0

^a^ Per-beneficiary spending for nonbevacizumab use was calculated as the weighted mean annual spending for beneficiaries using aflibercept or ranibizumab among ophthalmologists who did not accept any manufacturer payments. We assumed that if all ophthalmologists did not accept manufacturer payments, the nonbevacizumab use would reflect this distribution of aflibercept and ranibizumab use.

## Discussion

In this analysis of factors associated with variation in prescribing patterns for 3 ARMD treatments (bevacizumab, ranibizumab, and aflibercept), we found that payments from manufacturers of macular degeneration drugs to ophthalmologists were the largest factor in reduced bevacizumab use, exceeding the variations associated with patient or ophthalmologist characteristics. Manufacturer payments were associated with less prescribing of lower-cost therapies, which are factors in increased Medicare and patient spending. Controlling for patient and ophthalmologist characteristics, we found that ophthalmologists who accepted manufacturer payments prescribed lower-cost therapies nearly half as often as ophthalmologists who did not accept manufacturer payments (28.0% vs 45.8%), consistent with prior research.^26,27,37^ Although we did not separately analyze whether payments originated from Regeneron Pharmaceuticals Inc (manufacturer of aflibercept) or Genentech Inc (manufacturer of bevacizumab and ranibizumab), ophthalmologists who accepted these payments overall had higher prescribing of ranibizumab over bevacizumab, suggesting that payments from Genentech Inc likely encouraged prescribing of the higher-cost drug. While only 25.7% of ophthalmologists accepted manufacturer payments, the increased use of higher-cost therapies among them increased Medicare spending on ARMD therapies by more than $642 million during the study period (2.0% higher). The implications of this finding may be limited for Medicare spending but not for individual patients. The choice to use a more expensive therapy over bevacizumab cost an additional $5231.35 per Medicare beneficiary per year. For beneficiaries without a Medigap policy to cover their 20% coinsurance, the out-of-pocket cost would be $1046.27.

Prior research^15,25,36^ reported an association between ophthalmologist use of lower-cost therapies and the race and ethnicity of patients, but we did not observe such an association. Suggesting that an association exists between patient dual-eligibility status and race and ethnicity may have confounded prior analyses. The present study found that ophthalmologists with a greater percentage of dual-eligible beneficiaries in their patient population were significantly more likely to prescribe bevacizumab. While dual-eligible beneficiaries themselves generally do not experience Medicare cost-sharing, an ophthalmologist with a higher percentage of dual-eligible beneficiaries may also see a greater percentage of beneficiaries with income near the dual-eligible threshold, leading to prescribing choices that minimize patient cost-sharing. Ophthalmologists with longer practice duration were also significantly more likely to prescribe bevacizumab. This observation may reflect the continued use of off-label bevacizumab as a practice pattern that predated the introduction of ranibizumab and aflibercept.

Ophthalmologists who saw patients with a higher risk score were significantly less likely to prescribe bevacizumab, which may reflect ophthalmologist concern about the modest clinical differences between the 3 drugs for sicker patients. Similarly, ophthalmologists with older patients, on average, were significantly less likely to prescribe bevacizumab, which may also reflect clinical differences in therapy choice. Additionally, we observed substantial prescribing variation by MAC region, which may reflect the differences in patient populations, regional prescribing patterns, or MAC reimbursement policies.

Although this analysis estimated only the cost implications of manufacturer payments to ophthalmologists, its results have important implications for payment reforms. Even controlling for characteristics that may play a role in treatment selection (beneficiary age and risk score), we observed differences in treatment selection on domains that should not be factors (beneficiary dual-eligible status and ophthalmologist practice duration). If all ophthalmologists prescribed ARMD treatment similar to those ophthalmologists who saw more dual-eligible patients or those ophthalmologists with longer practice duration, substantial reductions in Medicare and patient spending could be the outcome. This outcome could be achieved by establishing a weighted manufacturer payment amount for ARMD therapy based on the utilization mix of the ophthalmologists who tend to use lower-cost therapies, adjusting for beneficiary risk score and age. This approach would be similar to the approach used for Medicare reimbursement of inpatient treatment, in which weighted mean costs are used to reimburse ophthalmologists and create a financial incentive for selecting the lower-cost treatment options.^17^ This approach could counteract the current incentive in the Medicare Part B program, where ophthalmologists earn greater add-on revenue from selecting a higher-cost therapy. A related approach was proposed by the Medicare Payment Advisory Commission, establishing a consolidated billing code with a single reference price for treatments with similar therapeutic outcomes, with consideration of ARMD treatments as well as rheumatoid arthritis therapies, prostate cancer treatments, leukocyte growth factors, and immune globulins.^17^ The Medicare Payment Advisory Commission reported that if the lowest-priced product (bevacizumab) were used to establish this reference price (a least-costly alternative policy), Medicare spending on ranibizumab and bevacizumab from 2008 to 2009 would have been $1.1 billion lower, with a $275 million reduction in beneficiary costs.^17^ Given the recent approval of a biosimilar formulation of bevacizumab, manufacturer payment models should incorporate the lower prices associated with this new formulation to further reduce ARMD treatment costs. Furthermore, extending this approach to other therapeutic areas could generate greater savings and encourage more value-based prescribing.

### Limitations

This study has several limitations. First, differences in billing for off-label bevacizumab use may affect the estimates; however, the sensitivity analysis that excluded all ophthalmologists who used nonbevacizumab-specific codes yielded consistent results. Second, the patient characteristics reflected the entire patient mix seen by the ophthalmologist, not just those treated for ARMD. If patients being treated for ARMD differ from the broader population, this could affect our estimates of the association between patient characteristics and ophthalmologist treatment selection. However, we assumed that any such differences were likely uniform across ophthalmologists and would not change the directional patterns observed. Third, we were unable to account for practice-level outcomes that may be associated with an ophthalmologist’s choice (eg, practice ownership structure, and bulk purchasing of a single drug by a practice group) due to data limitations. Fourth, this analysis was observational in nature.

## Conclusions

To our knowledge, this cross-sectional study was the first to estimate the increase in Medicare spending on ARMD therapies associated with pharmaceutical manufacturer payments to ophthalmologists. Ophthalmologists who accepted manufacturer payments were significantly more likely to prescribe higher-cost therapies. Had these ophthalmologists practiced prescribing similar to those who did not accept manufacturer payments, Medicare would have reduced spending on ARMD therapies by more than $642 million from 2013 to 2019. Additionally, ophthalmologists with fewer dual-eligible patients were also significantly more likely to choose higher-cost therapies, which may be associated with whether patients had supplemental Medigap coverage that insulated them from higher cost-sharing. Given that ophthalmologists appeared to gain greater profits when choosing higher-cost therapies, policymakers should counter this incentive by developing manufacturer payment models that encourage ophthalmologists to choose lower-cost therapies.

## Notes

Supplement.
Data Sharing Statement
