# Supplementary material for: Medicare Part B Spending on Macular Degeneration Treatments Associated With Manufacturer Payments to Ophthalmologists
Source: JAMA Health Forum. 2023 Sep 8;4(9):e232951. doi: 10.1001/jamahealthforum.2023.2951 (PMC10492178; doi:10.1001/jamahealthforum.2023.2951)
Supplement: Supplement. — Data Sharing Statement [file jamahealthforum-e232951-s001.pdf]

Data Sharing Statement

Dickson. Medicare Part B Spending on Macular Degeneration Treatments Associated With Manufacturer Payments to Ophthalmologists. *JAMA Health Forum*. Published September 08, 2023. doi:10.1001/jamahealthforum.2023.2951

Data
Data available: No

Additional Information
Explanation for why data not available: All data is publicly available
